# Nitric Oxide Enhancing Resistance to PEG-Induced Water Deficiency is Associated with the Primary Photosynthesis Reaction in *Triticum aestivum* L.

**DOI:** 10.3390/ijms19092819

**Published:** 2018-09-18

**Authors:** Ruixin Shao, Huifang Zheng, Shuangjie Jia, Yanping Jiang, Qinghua Yang, Guozhang Kang

**Affiliations:** Collaborative Innovation Center of Henan Grain Crops and State Key Laboratory of Wheat and Maize Crop Science/College of Agronomy, Henan Agricultural University, Zhengzhou 450001, China; zhenghuifang@gmail.com (H.Z.); jiashuangjie2018@gmail.com (S.J.); jiangyanping.up@gmail.com (Y.J.)

**Keywords:** nitric oxide, 20% PEG-induced water deficiency, phosphoproteomic, *Triticum aestivum* L., primary reaction of photosynthesis

## Abstract

Photosynthesis is affected by water-deficiency (WD) stress, and nitric oxide (NO) is a free radical that participates in the photosynthesis process. Previous studies have suggested that NO regulates excitation-energy distribution of photosynthesis under WD stress. Here, quantitative phosphoproteomic profiling was conducted using iTRAQ. Differentially phosphorylated protein species (DEPs) were identified in leaves of NO- or polyethylene glycol (PEG)-treated wheat seedlings (D), and in control seedlings. From 1396 unique phosphoproteins, 2257 unique phosphorylated peptides and 2416 phosphorylation sites were identified. Of these, 96 DEPs displayed significant changes (≥1.50-fold, *p* < 0.01). These DEPs are involved in photosynthesis, signal transduction, etc. Furthermore, phosphorylation of several DEPs was upregulated by both D and NO treatments, but downregulated only in NO treatment. These differences affected the chlorophyll A–B binding protein, chloroplast post-illumination chlorophyll-fluorescence-increase protein, and SNT7, implying that NO indirectly regulated the absorption and transport of light energy in photosynthesis in response to WD stress. The significant difference of chlorophyll (Chl) content, Chl a fluorescence-transient, photosynthesis index, and trapping and transport of light energy further indicated that exogenous NO under D stress enhanced the primary photosynthesis reaction compared to D treatment. A putative pathway is proposed to elucidate NO regulation of the primary reaction of photosynthesis under WD.

## 1. Introduction

Nitric oxide (NO) is a small gaseous signaling molecule that has attracted significant interest. As a bioactive molecule, NO is involved in numerous physiological processes in animals, and has also been reported as a mediator of both biotic and abiotic stress responses in plants [1]. Recently, various enzymatic and nonenzymatic pathways for its synthesis have been reported [2,3]. Important advancements have been achieved to elucidate the roles of NO in plant growth and development. The role of NO as a signal molecule has been established as an activator of reactive oxygen species (ROS), scavenging enzymes under abiotic stress [4,5,6,7]. Interestingly, NO can be rapidly induced by environmental stimuli, and also acts as a secondary messenger during environmental stress-related signal transduction [4,8]. Exogenous NO, supplied to plants via sodium nitroprusside (SNP) or potassium nitrite, can also improve plant tolerance for environmental stress.

Water deficiency (WD) is an environmental factor that significantly impairs both crop growth and productivity [9]. Recent trends in climate change have increased the frequency and severity of WD stress, indicating it as one of the main environmental issues of global concern [10]. Plants grown under WD conditions have evolved various mechanisms to resist WD stress. One vital adaptation is the regulation of photosynthesis as a countermeasure to oxidative stress arising in WD conditions. Some of these adaptations involve NO [11,12,13].

Understanding the complex effect of NO on plants requires a detailed analysis of both physiological and molecular changes. The adaptability of plants to WD at the cellular and physiological levels is implemented by either induction or repression of relevant genes. Recently, a large number of analyses of plant responses to NO have been conducted using different techniques: transcriptional analyses identified 510 NO-related genes in *Arabidopsis thaliana* [14]; comparative proteomics identified 92 NO-related proteins in *Oryza sativa*, indicating that exogenous NO alleviated Al^3+^ toxicity [15]; and 166 proteins were identified in *Anemone vitifolia Buch* using label-free quantitative proteomics after NO treatment without stress [16]. The above-identified NO-related genes or proteins were mostly involved in photosynthesis, stress, signaling, and secondary metabolism. NO has also been reported to be either directly or indirectly involved in the regulation of translation, and in post-translational modifications (PTMs). An established example is phosphorylation, which is one of the most prevalent and functionally important PTMs [17]. Phosphorylation is also a frequent post-translational modification because thousands of kinase genes occupy 3%–4% of functional genes in plants. Phosphorylation has therefore been widely employed to investigate the molecular mechanism of biotic/abiotic stress and the resulting hormone response.

Wheat (*Triticum aestivum* L.) is one of the most-produced cereal crops. Although previous studies indicated that NO can alleviate WD stress, no systematic profiling studies by “omics” have to date been published that dissect the effect of NO on wheat plants in response to WD stress. Here, comparative phosphoproteomics were performed to analyze the phosphorylation dynamics in response to exogenous NO donors (SNP) and 20% polyethylene glycol (PEG) 6000-induced WD (D) in wheat seedlings. Based on most protein species and in combination with the physiological level, NO-induced WD resistance was further verified, and a possible mechanism was suggested.

## 2. Results

### 2.1. Morphological Change in Response to NO and D Stress

**Figure 1:**
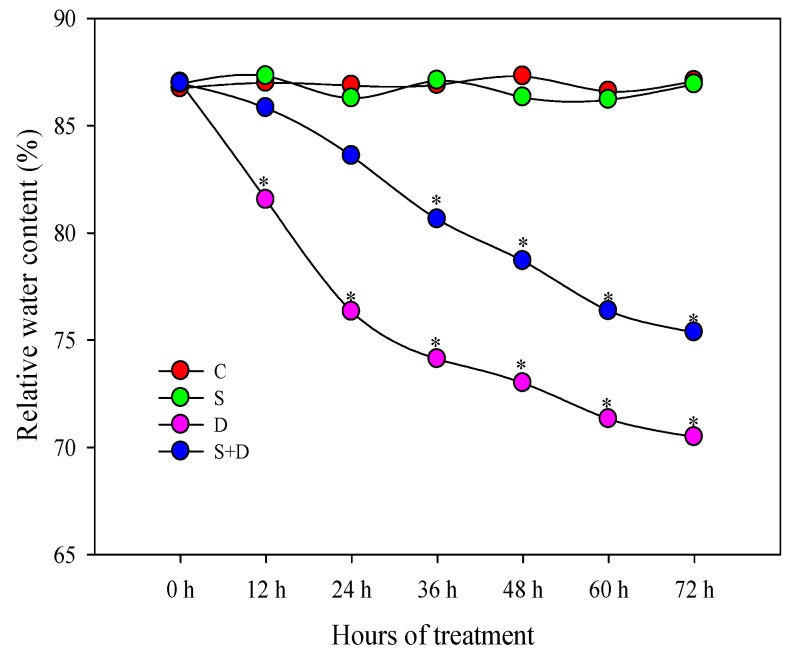
Change of relative water content (RWC) in leaves of wheat seedlings under normal water conditions and in response to three different treatments at different time points; a total of three independent biological replicates were conducted (*n* = 10). C, normal water conditions; S, pretreated with 150 µmol/L sodium nitroprusside; D, water deficiency induced by 20% polyethylene glycol (PEG)-6000; S + D, pretreated with 150 µmol/L sodium nitroprusside and water deficiency stress by 20% PEG-6000. Asterisks indicate significant differences among the four treatments at *p* < 0.05.

**Figure 2:**
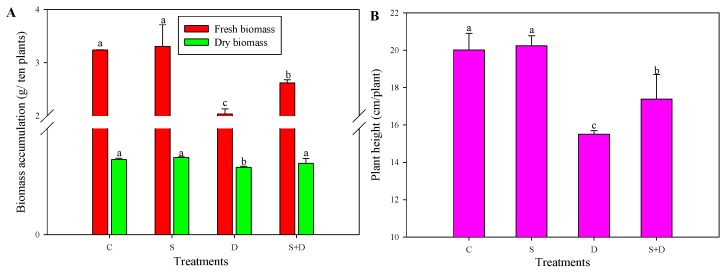
Phenotypic (**A**,**B**) changes in leaves of wheat seedlings under normal water conditions and in response to three different treatments; a total of three independent biological replicates were used (*n* = 10). For a detailed description of treatment conditions, please refer to the legend of Figure 1. Different lowercase letters indicate a statistically significant difference at *p* < 0.05.

Leaf relative water content (RWC) showed a gradual decline both in “D” and “S + D” treatments; however, “S + D” exhibited higher RWC than “D” plants (Figure 1) during 72 h of D stress. Biomass results showed that D without SNP treatment inhibited the growth of wheat, while the growth state in the “S + D” treatment exceeded that of the “D” treatment. Here, this was also reflected in fresh and dry biomass accumulation as well as in plant height; S + D increased by 28.8%, 6.0%, and 12.2%, respectively, compared to D treatment (Figure 2A,B).

### 2.2. Quantitative Identification of Phosphoproteins Using iTRAQ

**Figure 3:**
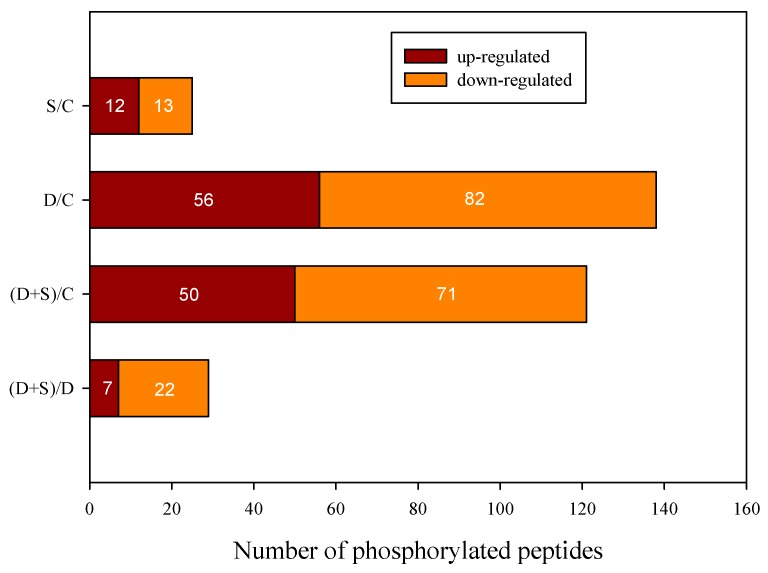
Upregulated and downregulated significant phosphorylated peptides among four groups. For a detailed description of treatment conditions, please refer to the legend of Figure 1.

A total of 2257 unique phosphopeptides were identified among all four experimental groups (Table S1); these peptides originated from 1396 unique phosphoproteins (Table S2) searched in the *pooideae* Uniprot database, and 2416 phosphorylation sites, 2110 (87%) of which contained serine (Ser) residues (Figure S1 and Table S3). A fold-change value >1 indicates that the examined phosphorylated peptide is more abundant (suggesting its upregulation), while a fold-change value <1 indicates a less abundant phosphorylated peptide (suggesting its downregulation). A total of 96 differentially expressed phosphorylated proteins (DEPs) were identified in D/C consisting of 37 increased and 61 decreased proteins; (S + D)/C had less DEPs than D/C, including four less-increased and four less-decreased proteins (Table 1). However, only 25 and 24 DEPs were found in S/C and (S + D)/D. In total, the four groups had 148 DEPs with 172 differentially expressed phosphorylated peptides (DEPPs) (>1.5-fold change and *p* < 0.05, see Figure 3). The peptide-sequence and phosphorylation-site (probabilities > 75%), protein description, peptide score, and fold changes of 198 spots associated with NO or WD are provided in Table S4. At least one phosphorylated S/T-Q site in most DEPPs (85%) was found to be localized to the nucleus.

### 2.3. Gene ontology and Principle Component Analysis (PCA) Analysis of Differentially Expressed Phosphorylated Protein Species in Response to D Stress and NO

**Figure 4:**
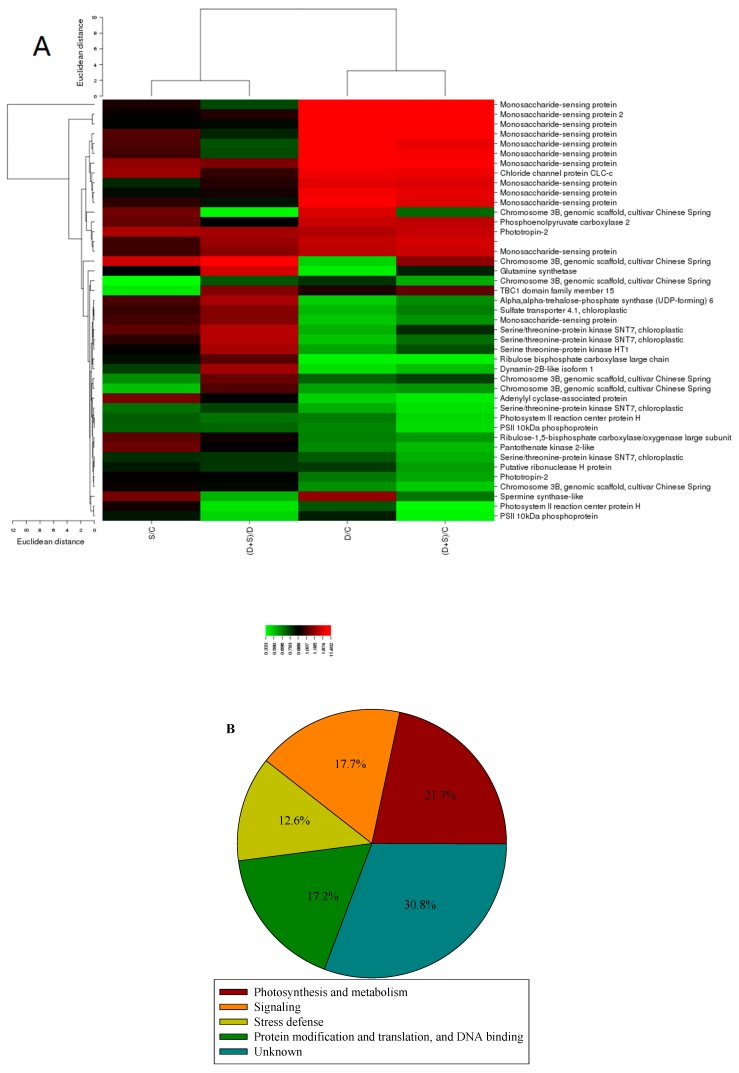
(**A**) Cluster analysis and (**B**) functional classification of significant differentially phosphorylated peptides in leaves of winter wheat among four different treatments. The color scale bar at the left of the hierarchical cluster analysis indicates the increased (red) and the decreased (green) peptides. For a detailed description of treatment conditions, please refer to the legend of Figure 1.

Hierarchical clustering analysis of DEPs detected different expression patterns in all four comparison groups (Figure 4A). Gene ontology (GO) analysis of DEPs indicated that most DEPs could be classified into 17 different biological processes. Fewer DEPs were found to have molecular function than those associated with cellular component and biological processes, and most DEPs were involved in binding. In biological processes, most DEPs were related to cellular processes, single-organism processes, biological regulation, metabolic processes, and response to stimulus (Figure S2). D/C or (S + D)/C exhibited two-fold increased DEPs compared to S/C or (S + D)/D, respectively (Figure S3). The function of the phosphorylated proteins listed in Table S4 was mainly divided into photosynthesis metabolism, signaling, stress defense, protein modification, translation, and DNA binding (Figure 4B).

PCA was performed to analyze the reason for the observed DEPs within the four groups. The variation explained by the first principle component (PC1, *x* axis) can be largely attributed to D; PC1 explains 50.6% of the variation. PC2 (*y* axis) explains an additional 10.8% of the variation, much of which is attributable to NO treatment (Figure S4). 

### 2.4. Metabolism-Related Differentially Expressed Phosphorylated Protein Species Accumulation in Response to NO and D Stress

Based on their classification, most DEPs (photosynthesis metabolism-related, 43 species) correlated with the photosynthesis metabolism (Table S4). No change in S/C was detected. Not surprisingly, most of the DEPs were downregulated when wheat seedlings suffered from D (D/C). However, some of these proteins were significantly upregulated (>1.5-fold change and *p* < 0.05) in (S + D)/D (Figure 4A), such as the chlorophyll a-b binding protein (Lhcb), chloroplast postillumination chlorophyll-fluorescence-increase protein (PIFI), and SNT7. The chosen DEPs are listed in Table 2, including their accession, coverage, proteins, unique peptides, Molecular weight, calc, and fold ratio. Only one peptide was phosphorylated with class II, the others were phosphorylated with class I. In these phosphorylation sites of class I, 11 DEPs have one or two phosphosites, located in either serine (Ser) or threonine (Thr) residue.

### 2.5. Physiological Changes of Photosynthesis-Related Parameters in Response to NO and D Stress

**Figure 5:**
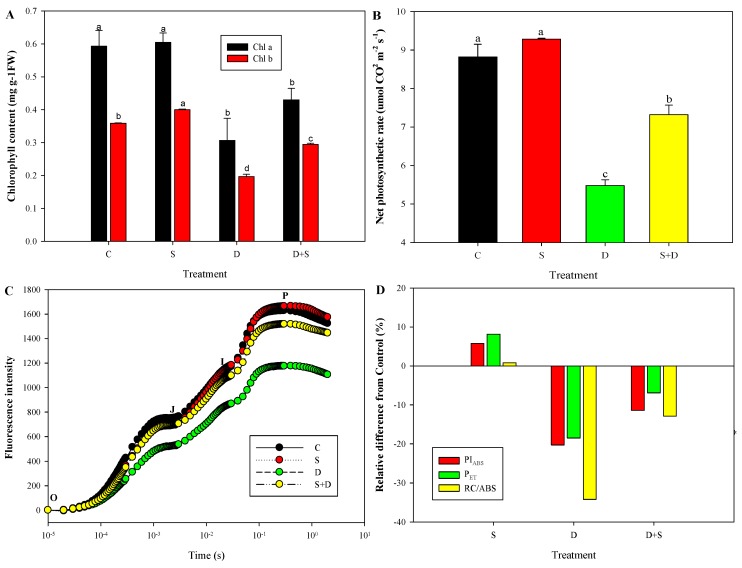
(**A**) Chlorophyll (Chl) content (Chl a, Chl b), (**B)** net photosynthetic rate (Pn), (**C**) fluorescence intensity, PI_ABS_, P_ET_, and (**D**) RC/ABS in leaves of wheat seedlings under normal water conditions and in response to NO or PEG-induced water deficiency treatments. C, normal water conditions; S, pretreated with 150 µmol/L sodium nitroprusside; D, water deficiency induced by treatment with 20% polyethylene glycol (PEG)-6000; S + D, pretreated with 150 µmol/L sodium nitroprusside and then water deficiency stressed by 20% PEG-6000. O, J, I and P mean that the analysis of the transient-considered fluorescence values at 50 ms (Fo, step O), 2 ms (F2 ms, step J), 30 ms (F30 ms, step I), and maximal level (FM, step P), respectively. For a detailed description of treatment conditions, please refer to the legend of Figure 1. Different lowercase letters in (**A**,**B**) indicate a statistically significant difference at *p* < 0.05.

Figure 5 shows the physiological changes associated with photosynthesis performance in response to both NO and D. No significant differences were found between C and S; however, D decreased by 48.2%, 45.1%, 37.87%, 34.2%, 20.3%, and 18.5% in Chl a, Chl b, Pn, RC/ABS, PI_ABS__,_ and P_ET_. When pretreated with SNP and then stressed by D, their values increased significantly; however, they could not completely recover to the control level.

## 3. Discussion

In abiotic stress studies, morphological, comparative physiological, and phosphoproteomic analyses are the most prevalent and effective strategies to investigate the mechanisms of abiotic stress resistance [18]. The results of the present study suggest that morphological and RWC changes after exogenous NO pretreatment contribute to D resistance. Furthermore, a comparative phosphoproteomic analysis among NO or D treatments was successfully performed. The results indicate that the phosphorylated peptides participated in key biological processes, which might work cooperatively to establish a new cellular homeostasis in response to NO.

### 3.1. Phosphoprotomics Elucidates Molecular Mechanism of NO-Induced D Tolerance in Wheat Seedlings

A number of studies indicated that phosphorylation played an important role in drought and chilling tolerance [17,18]. Here, 148 significant DEPs were identified in response to D or NO treatments. D was the single largest contributor to the variation among all four phosphoproteomic datasets obtained via PCA, followed by NO. This indicates that D treatment potentially impacts the phosphorylation of these proteins more significantly than NO treatment.

To date, quantitative phosphoproteomic studies in plants have shown that a majority of phosphoproteins only had one or two phosphorylation sites. Here, similar findings were obtained for wheat, and 76.9% of the phosphoproteins in four groups were found to possess fewer than two phosphorylation sites. However, only few DEPs were shown to have multiple phosphorylation sites. The distribution of phosphorylation Ser was maximal (87.3%) compared to the distributions of Thr and Lys, suggesting that these are preferentially highly sensitive and specific for Ser sites under D and NO treatment, followed by Thr and Lys residues. Identification of these phosphoproteins and phosphorylation sites provides a basis for the understanding of the molecular mechanisms underlying NO-regulated D resistance.

### 3.2. Comparison between Present Phosphoproteomic Data and Previous Studies

Only one NO-induced phosphoproteomic profile has been reported in cotton plants [19]; furthermore, no study on NO with a D stress-related phosphoproteomic profile has been published for higher plants. Two studies reported the D-induced phosphoproteomic profile in wheat [18,20]; however, one of these studies sampled wheat grains. Consequently, the results reported in these three References [18,19,20] were compared to the findings of this study. Thirty-four of the NO-induced phosphoprotein species found here were identical to those found by Fan et al. [19]; furthermore, nine and seven D–induced phosphoprotein species identical to those found here were identified in the remaining two studies [18,20], respectively. Differences and commonalities are listed in Table S5, most of which are related to photosynthesis metabolism and signaling; however, differential mechanisms could well be induced by NO with or without D stress in cotton or wheat. This difference is caused by different WD durations and extent, as well as wheat variety and sampling organization. The data reported herein aid the exploration of the molecular mechanism of NO-induced phosphorylation in response to severe D exposure.

### 3.3. Phosphorylation is Involved in the Photosynthesis Metabolism against D

Previous studies indicated that NO enhanced the stress resistance in other crops by regulating genes related to photosynthesis, metabolism, and stress signals [15,21]. Here, most of the phosphoproteins also participated in the photosynthesis metabolism, most of which in turn were downregulated after D treatment (Table 1, Figure S4). However, others were upregulated after NO plus D treatment, suggesting that the phosphorylation of these upregulated proteins was regulated by the interaction between NO and D; NO without D stress did not activate protein phosphorylation, which has also been reported before [19], suggesting that the effect of NO treatment is indirect.

Light-harvesting chlorophyll a/b protein (CAB) located in light-harvesting complex II (LHCII) can switch between light-harvesting antenna systems for photosystem (PS)I or PSII to maintain optimal excitation balance. This switch (termed state transition), involves the phosphorylation of CAB by specific thylakoid-bound Ser/Thr kinases (Stt7, SNT7, and TAKs) [22]. Polverari et al. [23] reported that NO modulated the CAB gene, SNT7 is required for the plastoquinone (PQ) redox state and provides a link between short- and long-term photosynthetic acclimation [24]. The novel nucleus-encoded chloroplast protein PIFI indicates the nonphotochemical reduction of PQ and has been reported to be involved in the NAD(P)H dehydrogenase complex-mediated chlororespiratory electron transport [25]. Here, the phosphorylation level at Ser or Thr sites showed significant decreases due to D stress, and no significant difference was found in response to NO exposure. However, phosphorylation of several peptides was significantly activated by the interaction of NO and D, indicating that, if these peptides are phosphorylated, they play an important role in the adaptation to D stress, which may be linked to the induction of NO.

### 3.4. NO-Enhanced D Tolerance Correlated with Primary Reaction of Photosynthesis

The central processes of photosynthesis are the absorption of light energy, electron transfer, and conversion of energy. Chl is an important pigment for photosynthesis and includes Chl a and Chl b. Chl a plays an important role as primary electron donor in the electron-transport chain and transfers resonance energy to the antenna complex, ending at the reaction center. Chl b aids the photosynthesis by absorbing light energy [26]. Chl a and Chl b concentrations influence the ability to absorb light energy. Numerous previous studies indicated that NO increased biomass accumulation and photosynthetic-performance driving force (PI_ABS_) due to both the trapping of excitation energy (RC/ABS) and electron transport (P_ET_) [2,5,12].

Here, changes of Chl a and Chl b content, Chl fluorescence intensity, PIABS, RC/ABS, and PET in response to NO and D treatment showed that absorption, transfer, and conversion of light energy were regulated by NO under D stress. The energy derived from the primary reaction of the photosynthesis is used in particular pathways to achieve the final result of photosynthesis [27].

### 3.5. Putative Mechanism of NO Regulates Primary Reaction of Photosynthesis in Response to D Stress

**Figure 6:**
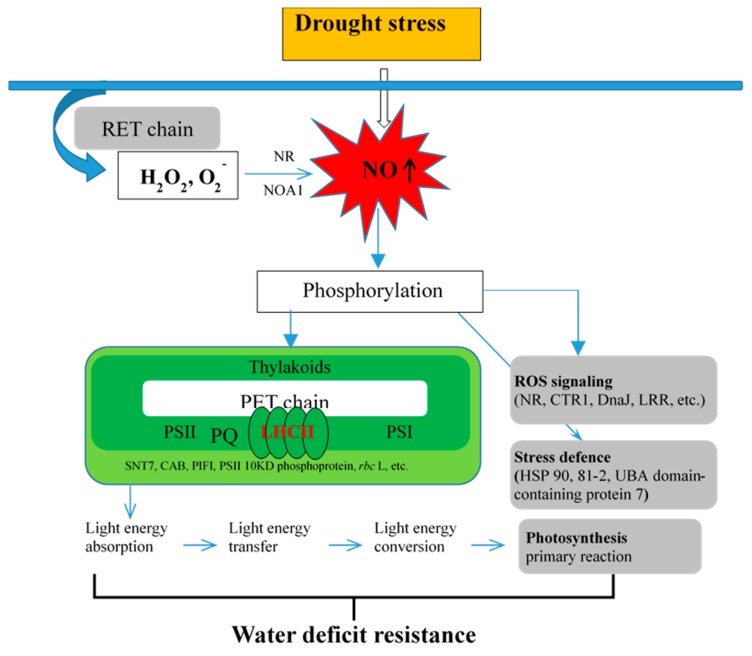
Schematic representation of NO involved in the regulation of photosynthesis to improve water-deficiency stress resistance. The contribution of NO to photosynthesis primary reaction under water deficit stress flows in the direction indicated by the arrow.

Combining the results reported here with those of previous studies [2,7,28,29,30,31] suggests that the primary reaction of photosynthesis may be regulated by NO involved in LHCII phosphorylation under D stress. The specific putative mechanism in wheat seedlings is shown in Figure 6.

In this study, in response to D stress, the ROS that was generated via oxidative burst (intersystem electron transport (PET) from chloroplast and respiratory chain (RET) of mitochondria) is suggested to trigger NO synthesis. Under D stress, NO activates the phosphorylation of photosynthesis, signaling, and stress defense-related peptides, thus promoting stress tolerance. Additionally, when seedlings suffered from D stimuli, PSII momentarily ran faster than PSI; thus, this led to the uneven excitation of both the PS-generated redox and ROS signals that are then transmitted as retrograde signals to the cytosol, mitochondrion, and nucleus. During PET, including the PQ pool, the LHCII kinase SNT7 activates the phosphorylated LHCII. For example, the CAB protein moved from PSII to PSI, compensates the imbalance, and upregulates the PIFI phosphorylation level. This improved absorption, transfer, and conversion of light energy. These changes therefore enhance the primary reaction of photosynthesis, which carries implications for the mechanisms with which NO improves WD resistance.

## 4. Materials and Methods

### 4.1. Plant Materials and D and NO Treatments

Seeds of common wheat cv. Zhoumai 18 were germinated for 14 h in glass petri dishes (15 cm) in an illuminated incubator (Laifu Technology, Ningbo, China). Standard growth conditions for wheat were used (14 h light: 10 h dark photoperiod; 25 °C day and 15 °C night; relative humidity of 60% during the day and 75% at night). Uniform seedlings were transferred to full-strength Hoagland’s liquid growth medium [32], and each petri dish contained approximately 60 seedlings. After 15 further days of growth, 3 dishes of each sample group were treated: for the C treatment, seedlings were incubated in Hoagland medium without SNP and PEG; for the D treatment, seedlings were incubated in Hoagland medium supplemented with 20% PEG (6000) (−0.8 MPa osmotic potential) (Sigma Chemical Co., St. Louis, MO, USA); for the S treatment, seedlings were incubated in Hoagland solution supplemented with 150 µmol/L SNP (Sigma Chemical Co., St. Louis, MO, USA); for the S + D treatment, seedlings were incubated in Hoagland medium supplemented with 150 µmol/L SNP for 3 days before also adding 20% PEG solution. At all stages of the experiment and for all sample groups, liquid media were changed daily. After 3 days of 20% PEG treatment, the uppermost fully developed leaves of all 4 treatments were collected, immediately frozen in liquid nitrogen, and stored at −80 °C until further physiological and phosphorylated proteomic analysis.

### 4.2. Phenotypic Analysis

Ten individual wheat seedlings were randomly harvested from each dish. The RWC in the leaves was determined with the method described by Wu and Xia [33]. RWC percentage was calculated using the following formula: RWC (%) = 100 × (FW − DW)/(SW − DW), where FW represents fresh weight, DW represents dry weight, and SW represents saturated weight. SW was determined after letting the leaflet float on distilled water for 24 h at room temperature.

Plant height and plant biomass (fresh and dry weights) were measured. Samples were then placed in an oven at 105 °C for 15 min followed by drying to a constant weight at 75 °C.

### 4.3. Physiological Analysis

Chl was extracted using the nonmaceration method developed by Hiscox and Israelstam [34]. Leaf samples (0.05 g) were incubated in 5 mL dimethyl sulfoxide at 65 °C for 4 h. Absorbance of the chloroplast pigments was measured at both 645 and 663 nm, and the Chl a and Chl b contents were calculated accordingly [35].

Fast Chl fluorescence-induction kinetics were measured using a Handy PEA Chl fluorimeter (Hansatech Instruments Ltd., Norflok, UK). Induced by red saturating light, the OJIP rising transients were measured with leaves that had been dark-adapted for 20 min. The analysis of the transient-considered fluorescence values at 50 ms (Fo, step O), 2 ms (F2 ms, step J), 30 ms (F30 ms, step I), and maximal level (FM, step P). On a logarithmic time scale, the increasing transient from Fo (F measured at 50 ms) to FP (where FP = FM under saturating excitation light, of which the excitation intensity was sufficiently high to ensure the closure of all RCs of PSII) showed polyphasic behavior. The JIP test represents a translation of original data into biophysical parameters that quantify the energy flow. The absorption of light energy (ABS), RC/ABS = ((F2 ms − Fo)/4(F300 ms − Fo))(Fv/FM) represents the active RC density on a Chl basis. A decrease in RC/ABS indicates an increase in the size of the Chl antenna serving each RC [2]. The contribution of dark reactions was calculated with the formula (ψo/(1 − ψo)) = (FM − F2 ms)/(F2 ms − F50 ms). The performance index of photosynthesis (PI_ABS_) represents the driving force of the performance. One component is performance due to the conversion of excitation energy into electron transport (P_ET_). The net leaf photosynthetic rate (Pn) was measured using a Li-Cor 6400 photosynthetic system (Li-Cor, Lincoln, NE, USA).

To dissect the impact of NO on the growth of wheat under WD, the second newly expanded leaves from 3 treated and untreated (Control) samples were harvested for protein extraction, iTRAQ labeling, and identification of phosphorylated peptides.

### 4.4. Protein Extraction

For each sample group, 5 g of tissue from each of the 3 biological replicates were ground with a mortar and pestle and transferred into chilled 50 mL conical tubes. Protein extraction was conducted according to Pi et al. [36].

### 4.5. Protein Digestion and iTRAQ Labeling

Protein digestion was performed using a method involving filter-aided sample preparation (FASP Digestion) [37]. After the resulting peptides were collected by centrifugation at 14,000× *g* for 10 min, the filters were then rinsed with 40 µL 106 DS buffer (50 mM triethylammonium bicarbonate at pH 8.5) and centrifuged a final time. Peptide concentration was estimated by UV light spectral density at 280 nm using an extinctions coefficient of 1.1 of 0.1% (g/L) solution that was calculated on the basis of the frequencies of tryptophan and tyrosine in vertebrate proteins. For iTRAQ labeling, 100 μg of sample peptides was separately labeled for each biological replicate using an iTRAQ Reagent4plex Multiplex Kit (AB Sciex, Framingham, USA). 114, 115, 116, and 117 labels were used for the C, S, D, and S + D sample groups, respectively. Labeling was conducted according to the manufacturer’s instructions.

### 4.6. Enrichment of Phosphorylated Peptides Using TiO_2_ Beads

The labeled peptide samples were concentrated using a vacuum concentrator and resuspended in 500 µL buffer (2% glutamic acid, 65% acetonitrile, and 2% trifluoroacetic acid). Then, 500 µg TiO_2_ beads (GL Sciences, Tokyo, Japan) was added, and samples were agitated for 40 min before centrifugation for 1 min at 5000× *g*. The supernatant was then discarded. The beads were washed 3 times with 50 µL 30% acetonitrile and 3% TFA and 3 times with 50 µL of 80% acetonitrile and 0.3% trifluoroacetic acid to remove the remaining nonadsorbed material. Finally, the phosphopeptides were eluted with 50 µL of elution buffer (40% acetonitrile, 15% NH_4_OH) and lyophilized [38]. Each iTRAQ sample (5 µL) was mixed with 15 µL of 0.1% trifluoroacetic acid (*v*/*v*) for mass spectrometry analysis.

### 4.7. Liquid Chromatograph -Mass Spectrometry Analysis

A 5 µL mixture from each sample was added to a Thermo Scientific EASY precolumn (2 cm × 100 μm × 5 μm C18) in buffer A (0.1% formic acid) and separated with a linear gradient of buffer B (80% acetonitrile and 0.1% formic acid) at a flow rate of 250 nL/min on a Thermo Scientific ||instrument||nLC (ProxeonBiosystems, now Thermo Fisher Scientific, Waltham, MA, USA) EASY column (75 μm × 250 mm × 3 μm C18). The peptides were eluted with a gradient of 0–55% buffer B from 0 to 220 min, 55–100% buffer B from 220 to 228 min, and 100% buffer B from 228 to 240 min.

Following nano-Liquid Chromatograph (LC) separation, Mass Spectrometry (MS)/MS analysis was performed on a Q exactive mass spectrometer (Thermo Fisher Scientific). The mass spectrometer was operated in positive-ion mode. MS data were acquired using a data-dependent (top-10) method that dynamically selects the most abundant precursor ions from a survey scan (350–1800 *m*/*z*) for HCD fragmentation and uses the automatic gain control function as target value. The dynamic exclusion duration was 30.0 s. Survey scans were acquired at a resolution of 70,000 at *m*/*z* 200, and the resolution for the HCD spectra was set to 17,500 at *m*/*z* 200. Isolation width was set to 2 *m*/*z*. Normalized collision energy was 29 eV, and the underfill ratio (which specifies the minimum percentage of the target value likely to be reached by the maximum fill time), was set to 0.1%. The instrument was operated with enabled peptide recognition mode. Each iTRAQ experiment was analyzed 3 times.

### 4.8. Data Workflow

MS/MS spectra were searched using Mascot 2.2 (Matrix Science, Boston, MA, USA) and Proteome Discoverer 1.4 software (Thermo Fisher Scientific,) against both the *pooideae* Uniprot database (294, e962 sequences, downloaded 9 November 2015) and a decoy database. For protein identification, the following parameters were used: fragment mass tolerance, 0.1Da; peptide mass tolerance, 20 ppm; enzyme, trypsin; max missed cleavages, 2; fixed modifications, carbamidomethyl (C), iTRAQ 4plex (N-term), iTRAQ 4plex (K); variable modifications, oxidation (M), phosphorylation (ST), phosphorylation (Y). The score threshold for peptide identification was set at a false-discovery rate (FDR) of ≤0.01. Phosphorylated sites on the identified peptides were assigned using the PhosphoRS algorithm; for each phosphorylation site on all phosphopeptides, PhosphoRS probabilities above 75% were interpreted to indicate that a site is truly phosphorylated, and PhosphoRS scores above 50% were considered to indicate good peptide spectral match [39,40]. Please note that the expression change of phosphorylated peptides can be attributed to either the change in abundance of the corresponding protein, the phosphorylation level of the protein, or both; in this study, we did not attempt to dissect the relative contributions of these two factors. All datasets were processed using inhouse Perlscripts.

Phosphopeptide quantification was evaluated based on the intensity of the reporter ions. We normalized the phosphopeptide ratios by dividing the value for a given peptide by the median ratio of all identified peptides. The log2 fold-change values (S/C; D/C; (S + D)/C; (S + D)/D) for each treatment were calculated for each phosphopeptide. Only phosphopeptides detected in at least 2 out of the 3 biological replicates were used for the assessment of significant changes.

### 4.9. Identification of Differentially Phosphorylated Peptides

Differentially phosphorylated peptides were identified on a per-peptide basis and in a pairwise manner. Briefly, the abundance values for a particular phosphorylated peptide from 3 biological replicates of each experimental condition were compared to the abundances of the same peptide from a different experimental condition using a 2-sample Student’s *t*-test with default parameters. The peptide was considered to be differentially phosphorylated if the reported 2-sided *p*-value was below 0.05. The fold-change was then calculated for the peptide as the ratio between the mean value of the biological replicates of one experiment and the mean value of the other.

Multiple distinct phosphorylated peptides can be mapped to the same protein. Our analyses showed that these multiple peptides exhibit similar trends, i.e., they are either all significantly upregulated or all significantly downregulated between two experimental conditions, highlighting the accuracy and high quality of our data. To gain indepth insight of their phosphorylation patterns, we conducted hierarchical cluster analysis of the different phosphorylated peptides based on their phosphorylation intensities.

Additionally, PCA was performed using the built-in R function ‘prcomp’. PCA is often used to investigate the internal structure of the data in a way that best explains the variance in the data. In our case, PCA was used to illustrate the similarities among sample groups.

Accession numbers of sequence data can be accessed here: ProteomeXchange PXD010724.

### 4.10. Statistical Analysis

All experiments were repeated independently 3 times. Growth and physiological parameters were statistically analyzed using one-way analysis of variance (ANOVA) and Duncan’s multiple-range test to determine significant differences among group means. Significant differences from control values were determined at the *p* < 0.05 level.

## 5. Conclusions

In this study, 20% PEG greatly inhibited both growth and leaf RWC of wheat seedlings; however, NO could alleviate this PEG-induced inhibition. Phosphorylation has been widely employed to understand the molecular mechanism of biotic/abiotic stress and hormone responses. Here, quantitative phosphoproteomic analysis was performed using iTRAQ in seedlings treated with S, D, and S + D compared with control. In total, 2257 unique phosphopeptides, 1396 unique phosphoproteins, and 2416 phosphorylation sites were identified among four experimental groups. In total, the four groups contained 148 DEPs with 172 different expressed DEPPs (>1.5-fold change and *p* < 0.05, Figure 3). Most species were involved in both photosynthetic metabolism and signaling. Most DEPPs decreased in response to D stress. However, several were increased after NO plus D treatment, such as part of the peptides of CAB, PIFI, and SNT7, which may be closely related to the absorption of light energy in the LHCII antenna and electron transport, as a primary reaction of photosynthesis. We suggest that the NO signal could play an important role in the phosphorylation of these peptides. The corresponding physiological change further confirmed the effect of NO on the primary reaction of photosynthesis in wheat seedlings suffering from D stress. This study provides insight into how the molecular mechanisms of NO improving D resistance in higher plants.

## Figures and Tables

**Table 1:** Number of differentially expressed significant phosphorylated proteins in the leaves of wheat seedlings.

Groups	Increase	Decrease	Each Total	Total Phosphorylated Proteins
S/C	12	13	25	148
D/C	37	61	96	
(S + D)/C	33	57	88	
(S + D)/D	7	17	24	

Notes: The rates of four groups exceeded +/−1.5, and *p* < 0.05. For a detailed description of treatment conditions, please refer to the legend of Figure 1.

**Table 2:** Phosphorylated peptides related to the primary photosynthesis metabolism that were differentially and abundantly accumulated in response to nitric oxide (NO) and D stress.

Description	Phosphosite ^a^	Accession	Coverage ^b^ (%)	Proteins ^c^	Unique Peptide ^d^	MW [kDa] ^e^	Calc. pI ^f^	Fold Ratio ^g^
								(S/C, D/C, (S + D)/C, (S + D)/D)
Lhcb	S(11): 94.0	F2CRC1	12.24	4	2	26.72	6.11	0.73/0.22/0.27/1.23 ↑
SNT7	T(1): 100.0; S(5): 100.0	M8CIW2	2.59	1	2	68.90	9.01	1.04/0.62/0.82/1.32 ↑
	T(4): 100.0; S(8): 100.0							0.95/0.59/0.75/1.26 ↑
	S(5): 100.0							0.82/0.76/0.62/0.82
	S(5): 100.0							0.74/0.63/0.49/0.78
PIFI	Class II	W5I170	14.98	3	1	24.69	4.77	0.79/0.60/0.72/1.20 ↑
	T(2): 100.0	B3TN78	19.18	1	1	7.83	8.53	0.75/0.69/0.52/0.75

Notes: Significantly upregulated phosphorylated peptides (*p* < 0.5) are marked with ↑. If they were downregulated in D/C, they have been restored after sodium nitroprusside (SNP) pretreatment ((S + D)/C) to a certain extent. ^a^ Potential phosphorylation sites of 0.75 or above that belong to class I are highly reliable; 0.75 > *p* ≥ 0.5 belongs to class II. Each line corresponds to the last column. S refers to serine, T refers to thrine. ^b^ Percentage of peptides assigned to the predicted protein. ^c^ Number of proteins in each proteome species. ^d^ Number of unique peptides assigned to each protein. ^e^ Molecular weight of proteins. ^f^ Isoelectric point of proteins. ^g^ Ratio of NO or drought-treated protein levels to control protein levels (S/C, D/C, (S + D)/C), NO plus drought-treated protein levels to drought-treated protein levels ((S + D)/D). Ratio corresponds to the group in the same column. For a detailed description of treatment conditions, please refer to the legend of Figure 1.

## Supplementary Materials

Supplementary materials can be found at http://www.mdpi.com/1422-0067/19/9/2819/s1.
