# Supplementary material for: Nitric Oxide Enhancing Resistance to PEG-Induced Water Deficiency is Associated with the Primary Photosynthesis Reaction in Triticum aestivum L
Source: Int J Mol Sci. 2018 Sep 18;19(9):2819. doi: 10.3390/ijms19092819 (PMC6164216; doi:10.3390/ijms19092819)
Supplement: Supplementary file 1 [file ijms-19-02819-s001.zip › Supplementary materials/Supplemental table 4.pdf]

Protein accession	Protein Description	Peptide Sequence	Peptide score	Fold change			
				S/C	D/C	(D+S)/C	(D+S)/D
**Photosynthesis and metabolism （43）**							
A1EA37	*Psb*H	ATQTVEDSSKPRPK	332.98	0.76	0.71	0.52	0.73
A1EA37	*Psb*H	ATQTVEDSSKPRPK	149.88	0.93	0.77	0.37	0.49
B3TN78	Chloroplast post-illumination chlorophyll fluorescence increase protein, putative	ATQTVEDSSKPKPR	291.88	0.75	0.69	0.52	0.75
B3TN78	Chloroplast post-illumination chlorophyll fluorescence increase protein, putative	ATQTVEDSSKPKPR	160.59	0.85	0.84	0.44	0.52
M7YY84	Inner membrane protein ALBINO3	ALGAGDSNGSTTSEDEESDDDTTEEGGPVEETFATGNDK	179.28	0.98	1.44	1.72	1.19
M8AUZ4;F2CR56	Phosphoenolpyruvate carboxylase 2	LSSIDAQLR	184.14	1.05	1.52	1.36	0.90
M8CIW2	SNT7, chloroplastic	IVRTISESMDELTSQR	413.24	0.95	0.59	0.75	1.26
M8CIW2	SNT7, chloroplastic	TISESMDELTSQR	230.02	0.82	0.76	0.62	0.82
M8CIW2	SNT7, chloroplastic	TISESMDELTSQR	335.24	0.74	0.63	0.49	0.78
M8CIW2	SNT7, chloroplastic	TISESMDELTSQR	317.91	1.04	0.62	0.82	1.32
P11383	*Rbc* L	FEFEPVDTIDK	349.38	0.86	0.45	0.47	1.04
Q7YMS7; P11383; A0A0A1IXX6; G0Y7R9;U5IKJ3	*Rbc* L	GGLDFTKDDENVNSQPFMR	213.71	1.04	0.69	0.65	0.93
R7WA66	Phototropin-2	NRLSENTEIQSAK	109.92	0.90	0.72	0.63	0.88
R7WF07	Phototropin-2	ADSPEREPPKASDADK	252.04	1.25	1.26	1.52	1.21
M7YMZ3	CSN5B subunits	ASASGTAPDSPVPEPMVEA	352.61	0.90	1.61	0.99	0.62

M8BRX5; T1N9G3	Putative ribonuclease H protein	STFFDDSVPSSPAYTSGFSPK	269.77	0.84	0.79	0.64	0.81
M8BXA3	Pantothenate kinase 2-like	GGVDSPSWR	179.35	1.05	0.68	0.60	0.89
M8C3K7	Alpha-alpha-trehalose-6-phosphate synthase	SYSNLLELAGGADR	358.61	1.01	0.54	0.66	1.22
C7DPL0;M7YLX8	Glutamine synthetase	WNYDGSSTGQAPGEDSEVILYPQAIFK	150.54	0.89	0.47	0.83	1.77
W6EK38	Spermine synthase-like	NVSAAAAQTEGSGDDGSR	123.49	1.06	1.19	0.73	0.61
M7YY67	DEAD-box ATP-dependent RNA helicase 3, chloroplastic	LGLGGGSDDEDDEGYGSDQEGPAVEGDADELAISR	412.43	1.28	1.59	1.94	1.22
M8B436	Endoglucanase 11	DLDVASPEEAPEAEEK	272.51	0.93	0.64	0.52	0.81
M8B436; M7ZLF7	Endoglucanase 11	IVESKPEFSTDEEQSPK	311.69	0.90	0.35	0.44	1.26
M8B436; M7ZLF7	Endoglucanase 11	NLPNDTDDLQSPLSTK	135.82	1.03	0.63	0.54	0.85
M7ZLF7	Endoglucanase 11	EPEFSTDEEESPK	132.07	1.03	0.35	0.45	1.29
A0A0A6ZFX8	Delta-1-pyrroline-5-carboxylate synthase-like	LINSSFADLQNPQLDLDGK	362.12	1.07	3.25	1.91	0.59
M8APZ9	Choline-phosphate cytidylyl transferase B	DFSLLQYDSDDFDDFEEEDDEVAK	373.64	1.03	2.09	1.88	0.90
M7ZU45	Protein phosphatase 2C, putative	GAQLSGPLDAAVPFSGPLPTKPTK	238.22	0.73	0.63	0.61	0.97
T1N9X5	Apocytochrome f precursor, putative	VQLYEMNF	117.21	0.80	0.59	0.57	0.96
W5I170	Chloroplast post-illumination chlorophyll fluorescence increase protein, putative	LDIVSGCMDPTSHMFDPLATIDDGSCPLDSDTEE	126.00	0.79	0.60	0.70	1.17
F2CRC1	Chlorophyll A-B binding protein	APVAALRPAASASSPR	135.03	0.73	0.22	0.26	1.18
M8AGD8	Monosaccharide-sensing protein 2	STLFPNFGSMFSVAEQQQAK	143.54	0.93	3.73	3.54	0.95
M8AGD8	Monosaccharide-sensing protein 2	STLFPNFGSMFSVAEQQQAK	175.63	1.17	2.21	2.42	1.10
M8AGD8	Monosaccharide-sensing protein 2	MPEVMGSMR	186.99	0.87	4.02	3.49	0.87
M8AGD8	Monosaccharide-sensing protein 2	QATSVEGK	249.39	0.94	11.40	8.92	0.78
M8AGD8	Monosaccharide-sensing protein 2	GGGQSALGSALGLMSR	381.32	0.86	2.12	1.99	0.94
M8AGD8	Monosaccharide-sensing protein 2	GGGQSALGSALGLMSR	389.65	0.83	1.94	1.85	0.95
M8AGD8	Monosaccharide-sensing protein 2	GGGQSALGSALGLMSR	336.15	1.01	2.58	2.00	0.78
M8AGD8	Monosaccharide-sensing protein 2	GGGQSALGSALGLMSR	222.25	0.96	2.20	1.87	0.85

M8AGD8	Monosaccharide-sensing protein 2	GGGQSALGSALGLMSR	390.17	0.98	2.77	2.15	0.78
M8AGD8	Monosaccharide-sensing protein 2	GGGQSALGSALGLMSR	390.17	1.03	2.87	2.37	0.83
M8AGD8	Monosaccharide-sensing protein 2	ADWDAESHRDDEDYASDHGADDIEDSLNSPLISR	271.21	0.98	1.36	1.56	1.15
M8AGD8	Monosaccharide-sensing protein 2	ADWDAESHRDDEDYASDHGADDIEDSLNSPLISR	209.12	0.98	0.59	0.65	1.11
**Signaling （35）**							
M7ZID8	Adenylyl cyclase-associated protein	GPPPPAAPSAPLFSTDKSPK	419.04	1.09	0.54	0.47	0.88
M7YDB1	Sulfate transporter 4.1, chloroplastic	LSPQASGNLTESPK	244.97	0.98	0.61	0.70	1.14
M8CTF4	Chloride channel protein CLC-c	YASSDALLR	202.97	1.20	2.11	2.02	0.96
N1R4E2	Dynamin-2B-like isoform 1	AAASSYSNDSPEAESPRTPGRPGEDWR	162.66	0.79	0.49	0.60	1.21
M7ZDI0	TBC1 domain family member 15	SLSSLELPGVATVANAMSR	241.40	0.48	0.94	1.04	1.10
M8BML2	Serine threonine-protein kinase HT1	VEDMDSAYGSDASDEGADDGDDLSVR	347.07	0.92	0.64	0.78	1.22
W5H319	Nitrate reductase	IGELITTGTGYNSDNSVHGGSSLSHLAPIR	406.03	1.02	0.42	0.60	1.41
W5H319	Nitrate reductase	STSTPFMNTVADK	147.19	0.86	0.34	0.42	1.24
W5H319	Nitrate reductase	STSTPFMNTVADK	305.58	0.94	0.38	0.49	1.30
W5H319	Nitrate reductase	VAGAPIALSSPR	131.50	1.13	0.59	0.66	1.12
M7YJJ8	MLO-like protein	SDDPQSAPTSPR	116.90	0.87	0.53	0.59	1.10
M7YSU4	Sugar transporter ERD6-like 6	KQPLLVNTGSWYR	226.39	0.82	2.07	2.10	1.01
A0A0C5B1N2	Methionine sulfoxide reductase MSRA	LGLGGGGSPR	258.07	1.03	0.68	0.62	0.91
B1B5D5	Ninja-family protein 3	TSSLPTETEEER	223.21	1.06	4.66	3.51	0.75
M7YMK0	Phosphoinositide phospholipase C 6	DGDAEQNPGKGTDDDAAWGTEVPDFK	224.85	0.98	0.66	0.84	1.26
M7ZNK2	Serine/threonine-protein kinase ATM	SLDSFEDLDAKSPTGGSFK	196.62	0.86	0.65	0.55	0.84
M7ZNK2	Serine/threonine-protein kinase ATM	SLDSFEDLDAKSPTGGSFK	181.11	0.96	0.84	0.62	0.74
M8CGA4	Serine/threonine-protein kinase CTR1	TSFLQPSDSGK	269.04	1.13	1.64	1.58	0.96
M8CGA4	Serine/threonine-protein kinase CTR1	ALSGGDGSSR	98.21	0.69	0.54	0.52	0.96
R7WAS5	Putative LRR receptor-like serine/threonine-protein kinase	LAPLPDTEGSAPGHVSTVVK	358.43	0.68	0.35	0.38	1.08
M8C0R9; W5GJY8	Serine/threonine-protein kinase CTR1	DFPISPSSAR	255.39	0.76	1.50	1.46	0.97
M7YEH3	Serine/threonine-protein kinase CTR1	EGPSSGPSTPR	95.80	0.91	0.68	0.58	0.86

M7YEH3	Serine/threonine-protein kinase CTR1	EGPSSGPSTPR	95.80	0.91	0.68	0.58	0.86
F2E0H6	CRAL/TRIO domain containing protein	VPTPDLEEAK	120.36	1.60	1.37	1.46	1.07
F2CQP4	Auxin-repressed protein	SAASTPPASPLGSTPPISPFAGGGGR	95.18	1.61	1.09	1.62	1.48
M0YI88	Heat shock protein DnaJ, putative	LLAAEGLELDTDEDDDDTTFLK	194.36	1.60	1.07	1.72	1.61
M0UVB7	Aquaporin protein, putative	FGSSASFGSR	85.24	1.10	0.67	0.61	0.92
M0UVB7	Aquaporin protein, putative	FGSSASFGSR	219.10	1.09	0.64	0.69	1.07
N1R4G7	Aquaporin protein, putative	LGSSASFGRN	169.76	0.81	0.55	0.56	1.06
M0VQA3	Transmembrane amino acid transporter protein, putative	AMAAMMITLAVITSSIAIASNVMSSINGEVK	373.60	0.43	0.49	0.89	1.83
M7ZEF9	Sodium/calcium exchanger protein, putative	VAASGAYSNK	213.16	0.82	2.67	1.16	0.43
M8BY61	Sodium/calcium exchanger protein, putative	VEASGAYSNK	187.48	0.96	2.38	0.98	0.41
M0VDL8	SNF1-related protein kinase regulatory subunit beta-2, putative	DDHEGVQVVGFEVPASPDSSYSNPIPGSEDEDR	233.46	1.48	2.02	0.99	0.49
M0VUF4	CAMK includes calcium/calmodulin depedent protein kinases	FTSLSLK	143.56	0.71	0.63	0.56	0.89
W5B7U0	Brassinosteroid insensitive 1-associated receptor kinase 1 precursor	AVELSGPR	127.50	0.82	0.58	0.65	1.13
**Stress defense（25）**							
M8BUS3	Sodium/hydrogen exchanger	SVHGPGLLGTVTEAEDR	281.73	1.21	1.43	1.54	0.69
F4Y592	Heat shock protein 90	EISDDEDEDEK	182.84	1.51	1.27	1.34	1.06
M7Z927	Glutathione peroxidase 4	ALEEEPSDSQTK	293.22	1.27	1.52	1.14	0.75
A1IVL7	Dehydrin 2 (Fragment)	SGSSSSSSSEDDGMGGR	425.33	1.67	11.57	3.50	0.30
A1IVL7	Dehydrin 2 (Fragment)	SGSSSSSSSEDDGMGGR	96.07	0.67	6.79	3.22	0.47
A1IVL7	Dehydrin 2 (Fragment)	SGSSSSSSSEDDGMGGR	362.12	1.43	7.44	2.31	0.31
M7YZ42	Endoplasmin-like protein	EVDVEVPADEESSEEEESTTETTEEEETEDDEEK	371.71	1.19	1.71	1.53	0.90
M7ZQA6	Heat shock protein 81-2	EISDDEDEEEK	361.37	1.65	1.28	1.38	1.10
M8BK61	Putative ubiquitin carrier protein E2 23	TENVGPDGDNANNASHGDDVDSQSSLPDNK	150.36	0.92	0.67	0.80	1.20
M8BUM2	UBA domain-containing protein 7	DIFPGAEPPASPRPGGASGDR	214.19	0.39	0.81	0.90	1.11

M8BUM2	UBA domain-containing protein 7	DIFPGAEPPASPRPGGASGDRDVFGAPTSPGGSTSR	261.79	0.39	0.45	0.49	1.09
M7ZZ93	Auxilin-related protein 2	SNVSAGSPR	71.99	1.06	0.59	0.59	0.99
M8BQI6	Chaperone protein dnaJ 10	NIDNMDDNAGSSPDSSPK	84.41	1.95	1.94	2.22	1.15
M8CDA0	DnaJ homolog subfamily C member 2	AAPSIVSPLSTR	177.21	0.86	0.65	0.60	0.93
F2D074;R7W122	Stem-specific protein TSJT1, putative	VDSQGQMCGSTFK	403.76	1.07	0.61	0.69	1.13
F2D074;R7W122	Stem-specific protein TSJT1, putative	VDSQGQMCGSTFK	234.71	1.05	0.66	0.71	1.08
R7W122	Stem-specific protein TSJT1, putative	VGSAADWSNHF	179.31	1.07	0.66	0.80	1.22
F2D074	Stem-specific protein TSJT1, putative	VGSAADWSNHY	286.45	1.06	0.58	0.67	1.14
R7W5G2	Ankyrin repeat-containing protein, putative	AGAAAPPVLVLSNSGKR	153.62	0.65	1.08	0.85	0.79
M8ADB8	Zinc finger family protein, putative	ATNLTSSPVSSPTAEDAR	57.89	1.39	1.60	2.32	1.45
M7YF60	DUF292 domain containing protein, putative	TPESPKSPAVSSR	208.79	1.11	0.63	0.63	1.01
R7WGK9	Arsenate reductase, putative	FVTVSSTSTPSRTSR	343.94	0.86	0.55	0.58	1.07
M8A7J8	Arsenate reductase, putative	FVTVSSTSTPSRTTR	270.10	0.91	0.53	0.51	0.96
M8A7J8; R7WGK9	Arsenate reductase, putative	LLPGSVDS	162.01	0.86	0.53	0.47	0.88
F2E4Q1	Universal stress protein domain containing protein	APTPRPPPPASLQPDSPGVFFTNAAAAAPLGSAHR	85.79	0.95	2.67	1.59	0.59
**Protein modification and translation, and DNA binding （34）**							
M7ZT14	Putative aquaporin PIP2-7-like	ALGSFRSTGAATAR	327.41	0.79	1.68	0.94	0.56
M7YJI2	Eukaryotic translation initiation factor 5A	SDSEDHHFESK	385.47	0.80	0.57	0.56	0.98
M7YJI2	Eukaryotic translation initiation factor 5A	SDSEDHHFESK	360.39	0.95	0.66	0.74	1.13
N1QZ68	Eukaryotic translation initiation factor 4G	SGVPYSPNR	215.68	0.77	0.61	0.59	0.96
R7W3V9	Eukaryotic translation initiation factor 5A	SDSEEHQFESK	155.21	0.80	0.65	0.70	1.07
R7WG18	Transcription factor RF2b	AASAAESSENEAESQSTSAER	157.01	1.41	2.81	1.67	0.59

M7ZED3	Rho guanine nucleotide exchange factor 6	VSPTHVHSNSISEDPALTETSEPTR	483.65	0.64	1.00	1.02	1.03
R7W3W8	Peptidyl-prolyl cis-trans isomerase	TPPAESVESEDEDGFPVSESK	195.52	0.91	1.60	1.42	0.89
M8A1W5	6-phosphofructo-2-kinase/fructose-2, 6-biphosphatase	SVGTLIQLQK	349.49	0.99	0.58	0.75	1.30
M8A1W5	6-phosphofructo-2-kinase/fructose-2, 6-biphosphatase	SFSACSLASGLNFGSTK	399.34	0.87	0.62	0.65	1.04
M8A1W5	6-phosphofructo-2-kinase/fructose-2, 6-biphosphatase	SFSACSLASGLNFGSTK	336.04	1.05	0.66	0.94	1.42
M8BG45	Putative TPR repeat-containing protein	EMNETDVPVSYSGTGIPEK	73.30	1.83	0.80	1.35	1.69
R7WBJ6	APOBEC1 complementation factor	HGGEDSDEPEEEPEEPQIEVEGDGDGDGR	96.07	1.44	1.64	1.47	0.90
M8AC57	U3 small nucleolar RNA-associated protein 18-like protein	SADDDLPVYEEDLGSEDEVLDK	109.19	0.84	1.51	1.39	0.92
M8BJ40	Heterogeneous nuclear ribonucleoprotein Q	VEEQKPQPSPPPQQPAAEVK	113.93	1.54	1.71	1.54	0.90
Q3S4I1	Eukaryotic translation initiation factor 5A	SDTDEHHFESK	367.76	0.78	0.66	0.63	0.96
F2CPU8; N1QPW1	Meiosis protein mei2	SPAPLSPPGFAVPLAVK	126.51	0.72	0.51	0.46	0.89
M8BA43	FACT complex subunit SSRP1-B	TGAVAAALQSTDDDPVDPHLER	87.00	1.97	1.87	1.97	1.05
M8CFC0	Putative WRKY transcription factor 20	SAHPDILPSPR	142.94	2.23	2.27	1.68	0.74
M7YDH3	Inositol hexakisphosphate and diphosphoinositol-pentakisphosphate kinase 2	SGQESDSDAEDLEHIEK	532.22	1.00	0.50	0.67	1.34
M8AL80	Brefeldin A-inhibited guanine nucleotide-exchange protein 2	IGLENSDGLPSPSGR	328.98	0.92	0.76	0.65	0.85
M8ATG2	Nuclear transcription factor Y subunit B-3	PDSDNEDSGNAGGELSSPR	227.93	1.21	0.34	0.45	1.33
A0A077S159	Chromosome 3B, genomic scaffold, cultivar Chinese Spring	SEAEVVVISPSEASPRSEAGESSEFQSEK	181.80	1.06	1.82	0.75	0.41
A0A077RQ44	Chromosome 3B, genomic scaffold, cultivar Chinese Spring	ALSATAHADQSPQDADPER	400.59	0.66	0.75	0.79	1.05

A0A077RU36	Chromosome 3B, genomic scaffold, cultivar Chinese Spring	AQIATVR	219.90	1.72	0.53	1.17	2.20
A0A077RT44	Chromosome 3B, genomic scaffold, cultivar Chinese Spring	AASKEPISPGSPSPASK	375.48	0.59	0.64	0.65	1.01
A0A077RWI8	Chromosome 3B, genomic scaffold, cultivar Chinese Spring	ILPQAEPVSSSPTAASGNR	426.80	0.93	0.66	0.57	0.87
A0A077S3J6	Chromosome 3B, genomic scaffold, cultivar Chinese Spring	SGSSTGSTSPGGVSPSHHR	119.48	0.33	0.82	0.63	0.77
M8B780	Bromodomain containing protein	WPEIEAEMAQLSPQPPTPSSAPPK	92.74	0.66	1.09	0.74	0.67
F2CXF2	KH domain containing protein	MEAPGSPYASSPDTAPK	153.98	0.86	0.60	0.67	1.11
M0Z854	FACT complex subunit SPT16, putative	DHGAESDSEEER	174.42	1.66	1.35	1.60	1.19
W5BP21	SNF2 family N-terminal domain containing protein, putative	NISYAESDSDDSEEK	230.91	1.25	1.74	1.59	0.91
M0XWV5	60S acidic ribosomal protein, putative	KEEAKEESDDDMGFSLFD	180.61	0.94	1.11	0.74	0.66
R7W0N2	CBS domain containing membrane protein, putative	EFDHINTVLSPTTPGSR	155.51	0.86	0.66	0.63	0.96
**Unknown （61）**							
M7Z3D7	Uncharacterized protein	TYETAQSAK	114.84	1.28	20.33	6.53	0.32
M0W1H7	Uncharacterized protein	EEPEIHADPAMDPNYESPEEPEEDAER	136.55	1.84	0.84	1.45	1.72
M7ZGT2	Uncharacterized protein	WDTQEISPDIVEISEDDSPPKNTDDR	157.01	1.14	1.37	1.58	1.16
M8BWZ1	Uncharacterized protein	SGTGSDEGGYNK	245.31	0.83	2.26	1.51	0.67
M8BWZ1	Uncharacterized protein	SSPDDYTGGGSYNK	310.31	1.03	2.33	1.85	0.80
M8BWZ1	Uncharacterized protein	SSIEEDYSSGKNTPNTDDYDGSGYNK	117.45	0.96	2.01	1.38	0.69
M8BWZ1	Uncharacterized protein	SSIEEDYSSGK	266.15	0.93	1.95	1.48	0.76
M8BWZ1	Uncharacterized protein	SSTDNFTGGLNK	128.19	1.04	1.80	1.71	0.95
M8BWZ1	Uncharacterized protein	KPSTEDSPAYNK	230.90	0.98	1.73	1.49	0.86
M8BWZ1	Uncharacterized protein	SSTDNFTGGLNKSSPDDYTGGGSYNK	252.80	0.58	2.04	1.55	0.76
M8C9W4	Uncharacterized protein	SAELGTEVGSPTGPVVR	342.30	0.70	0.63	0.57	0.91
M0XJF8	Uncharacterized protein	IMASEDENEDAEAPVIQK	172.16	0.86	0.56	0.71	1.27
M7ZGF0	Uncharacterized protein	AVSTGTLESPR	186.36	0.93	0.71	0.60	0.84

T1MPT4	Uncharacterized protein	LDYNTDDDFASAASPPNDVGPVGK	353.92	1.09	0.64	0.78	1.21
T1MPT4	Uncharacterized protein	LDYNTDDDFASAASPPNDVGPVGK	34.89	0.88	0.54	0.53	0.98
M8BNF9	Uncharacterized protein	SGSSGLVWDER	199.67	0.85	0.64	0.74	1.17
M8BTG2	Uncharacterized protein	AETPGSDNLK	461.65	0.58	0.59	0.58	0.98
M8BTG2	Uncharacterized protein	AETPGSDNLK	305.78	1.17	0.68	0.52	0.77
M8B9U9	Uncharacterized protein	HMPSQDSTNTSGDEEEGGR	250.93	1.60	2.05	1.97	0.96
M7ZK08	Uncharacterized protein	SDSIPLNSPPVKPTEQEFFTR	270.11	0.80	0.75	0.58	0.78
T1N701	Uncharacterized protein	TVAAADDEDTGGEENEGAQDVTDR	113.72	1.08	0.83	1.25	1.50
M8AXI3	Uncharacterized protein	VTRSEPSFTPAK	391.06	0.94	0.40	0.40	0.99
M8AXI3	Uncharacterized protein	SEPSFTPAK	215.73	0.98	0.40	0.47	1.16
M8AXI3	Uncharacterized protein	TSFSITSPK	100.48	1.17	0.65	0.74	1.14
M8AXI3	Uncharacterized protein	TSFSITSPK	172.00	1.05	0.59	0.77	1.31
M0W3G3; M8BFW3	Uncharacterized protein	AGNPAISSNSGENSPKPK	100.79	0.93	0.58	0.76	1.30
M0W3G3; M8BFW3	Uncharacterized protein	VPSNSSPRVSPNETAASVASSPLAK	187.56	1.06	0.69	0.59	0.86
M0W3G3; M8BFW3; T1MKZ8	Uncharacterized protein	SSSFNSK	136.70	1.05	0.56	0.80	1.43
M8BFW3; T1MKZ8	Uncharacterized protein	SCVQSPGAVQR	204.67	1.07	0.44	0.55	1.26
M0W3G3; T1MKZ8; M8BFW3	Uncharacterized protein	NKPVLPSGEVLPEAATSPK	381.13	0.93	0.62	0.82	1.33
T1MKZ8	Uncharacterized protein	AGNPTISSNSGENSPKPK	289.07	0.90	0.57	0.82	1.44
T1MKZ8	Uncharacterized protein	VPSNSSPRVSPNEAAASVASSPLAK	124.85	0.95	0.45	0.52	1.16
T1MKZ8	Uncharacterized protein	IGQNPSELADDEDQRSPPPNSDR	268.82	1.13	0.56	0.82	1.47
N1R2F7	Uncharacterized protein	GLVPVHGVGGSDR	113.27	1.41	1.57	2.26	1.44
M8ADA6	Uncharacterized protein	HNVPNDVPDESIEGPSDEEGEALAK	338.95	1.56	1.54	1.63	1.05
M8ADA6	Uncharacterized protein	HNVPNDVPDESIEGPSDEEGEALAK	148.99	1.78	1.73	2.40	1.39
M8AI93	Uncharacterized protein	LEQDGTPTTAISSGATLVDHGEANQGPFSPK	231.61	0.75	0.65	0.56	0.85

M8B807	Uncharacterized protein	SRSPASSPVAAGHEEIDAADAADAGK	252.33	0.93	0.67	0.52	0.78
M8BGL7	Uncharacterized protein	AAPVGSPKSPVK	128.75	0.98	0.66	0.54	0.82
M8BJM1	Uncharacterized protein	TTSLTEVAPPSVLAVVLEEEDEDEQVK	254.46	0.55	1.64	1.79	1.09
M8AYU0	Uncharacterized protein	LLLSQLSASR	168.60	0.90	0.58	0.55	0.94
M8B7V4	Uncharacterized protein	ELSIGDEVGWSK	254.45	0.97	1.71	1.37	0.80
M7Z6Q3	Uncharacterized protein	ETPNDDDDGTGSDGFELVDDVSPLTK	382.63	0.91	0.64	0.67	1.04
M7Z916	Uncharacterized protein	GLASSGSR	103.06	0.97	2.30	1.68	0.73
T1N801	Uncharacterized protein	SAPLPSLLSFTDSR	307.69	1.02	0.69	1.07	1.55
M8BVA8	Uncharacterized protein	DLIDPSIPTSPGPQSAPELDPNLPR	153.81	0.48	0.94	0.77	0.81
N1R3H2	Uncharacterized protein	QVDTLSPEEYSDEGWQAATLK	202.65	1.03	0.89	1.37	1.54
M7YYG1	Uncharacterized protein	FGGGYSSVLVPSPR	357.45	0.83	0.65	0.75	1.15
M7Z0P4	Uncharacterized protein	DSAAEETGVSEEYDGDETPSPETSTDEEVPR	154.42	0.90	1.60	1.79	1.12
M8BCL8	Uncharacterized protein	SDSIPLNSPQVKPTEQEFFTR	225.11	0.66	0.70	0.63	0.90
M7ZZM0	Uncharacterized protein	AQDSDVAKPSEVPLDNFSDDE	296.08	0.91	1.46	1.51	1.03
M8AEC2	Uncharacterized protein	AAAEHWGSPAAAAAFDADLVR	192.09	1.28	1.86	1.45	0.78
M8B0G2	Uncharacterized protein	SSPTSNLSPR	211.45	1.02	5.59	3.28	0.59
M8B0G2	Uncharacterized protein	SSPTSNLSPR	119.84	1.02	2.26	1.84	0.81
M7Z3N4	Uncharacterized protein	HAGSDSEEEGPVR	263.90	2.51	3.70	4.44	1.20
M7ZXM0	Uncharacterized protein	SSNAPVASGSPSNSVSK	96.05	1.53	1.45	1.75	1.21
T1M7A9	Uncharacterized protein	VESPTAGSTYGGGQQK	70.38	0.83	0.63	0.61	0.96
F2DL46	Uncharacterized protein	LYPASAGSSPSR	219.98	1.20	2.35	1.96	0.83
F2DF74	Uncharacterized protein	FGVIPSSPRTPMK	125.67	0.93	0.57	0.54	0.95
F2DMG4	Uncharacterized protein	SGSMDGASSPSDGESALSGGLPDYAK	464.61	2.04	1.82	1.87	1.03
F2CWB4	Uncharacterized protein	VDGPAPSAGGTASR	254.16	1.23	0.51	0.70	1.37
