# Supplementary material for: Nitric Oxide Enhancing Resistance to PEG-Induced Water Deficiency is Associated with the Primary Photosynthesis Reaction in Triticum aestivum L
Source: Int J Mol Sci. 2018 Sep 18;19(9):2819. doi: 10.3390/ijms19092819 (PMC6164216; doi:10.3390/ijms19092819)
Supplement: Supplementary file 1 [file ijms-19-02819-s001.zip › Supplementary materials/Supplemental table 5.pdf]

Treatments	Crop species and tissue	Common points	Difference	References
Nitric oxide	*Gossypium hirsutum,* leaf	1. Distribution of pS and phosphorylated Ser accounts for most of distribution and phosphorylated sites.<br>2. 34 common phosphorylated proteins were detected, such as CTR1, STN7, Phosphoenolpyruvate carboxylase 2, phototropin-2, Chloride channel protein CLC-c, Dynamin-2B, Monosaccharide-sensing protein 2, TBC1 domain family member 15, Serine/threonine-protein kinase HT1, Nitrate reductase, dehydrin, Heat shock protein 81, Nuclear Transcription factor Y subunit B-3, Aquaporin PIP2-7, Peptidyl-prolyl cis-trans isomerase, Sodium/hydrogen exchanger, Eukaryotic translation initiation factor 4G, DEAD-box ATP-dependent RNA helicase 3, Transcription factor RF2b, Sugar Ion/protein transporter ERD6-like 6, 6-phosphofructo-2-kinase/fructose-2,6-biphosphatase 1, TPR repeat-containing protein, Chaperone protein dnaJ, Meiosis protein mei2, Polyphosphoinositide phosphatase, LRR receptor-like serine/threonine-protein kinase, Inositol hexakisphosphate, Chlorophyll A-B binding protein, Stem-specific protein TSJT1, Ankyrin repeat-containing protein, Zinc finger family protein, Bromodomain containing protein, KH domain containing protein and diphosphoinositol-pentakisphosphate kinase.<br>3. There are no significant fold change in CTR1, STN7, Phosphoenolpyruvate carboxylase 2, phototropin-2, Chloride channel protein CLC-c, Dynamin-2B, Monosaccharide-sensing protein 2, TBC1 domain family member 15, Serine/threonine-protein kinase HT1, Nitrate reductase, DEAD-box ATP-dependent RNA helicase 3, dehydrin, Nuclear Transcription factor Y subunit B-3, Aquaporin PIP2-7, Eukaryotic translation initiation factor 4G, Transcription factor RF2b, Sugar Ion/protein transporter ERD6-like 6, 6-phosphofructo-2-kinase/fructose-2,6-biphosphatase 1, TPR repeat-containing protein, Meiosis protein mei2, Polyphosphoinositide phosphatase, Inositol hexakisphosphate, 60S acidic ribosomal protein and diphosphoinositol-pentakisphosphate kinase.	1. 34 up-regulated DEPPs and 50 down-regulated DEPPs were detected in the reference, but in this study only 12 up-regulated DEPPs and 12 down-regulated DEPPs were found.<br>2. LRR receptor-like serine/threonine-protein kinase, Stem-specific protein TSJT1, 60S acidic ribosomal protein and Chlorophyll A-B binding protein were up-regulated, and Peptidyl-prolyl cis-trans isomerase, Zinc finger family protein and Chaperone protein dnaJ were down-regulated in the reference.	Quantitative phosphoproteomics analysis of nitric oxide–responsive phosphoproteins in cotton leaf

		4. Heat shock protein 81, Sodium/hydrogen exchanger were up-regulated in both of two studies. Ankyrin repeat-containing protein, Bromodomain containing protein, KH domain containing protein were down-regulated in both of two studies.		
Drought	*Triticum aestivum* L., leaf	1. Three interesting function categories, such as signal transduction, RNA transcription/processing and stress defense stood out in both of studies.<br>2. 9 common phosphorylated proteins were detected, such as LRR receptor-like serine/threonine-protein kinase, Monosaccharide-sensing protein 2, TBC1 domain family member 15, Serine/threonine-protein kinase HT1, Peptidyl-prolyl cis-trans isomerase, FACT complex subunit SSRP1-B, Serine/threonine-protein kinase CTR1, Inositol hexakisphosphate and diphosphoinositol-pentakisphosphate kinase 2, Zinc finger family protein.<br>3. LRR receptor-like serine/threonine-protein kinase, Serine/threonine-protein kinase HT1, Inositol hexakisphosphate and diphosphoinositol-pentakisphosphate kinase 2 were down-regulated.<br>4. Monosaccharide-sensing protein 2 were up-regulated.	1. TBC1 domain family member 15, Zinc finger family protein were up-regulated in this reference.<br>2. Peptidyl-prolyl cis-trans isomerase, FACT complex subunit SSRP1-B were down-regulated in this reference. But it was up-regulated in this study.<br>3. There is no significant change of Serine/threonine-protein kinase CTR1 in this reference.	Phosphoproteome analysis reveals new drought response and defense mechanisms of seedling leaves in bread wheat (*Triticum aestivum* L.)
Drought	*Triticum aestivum* L., grains	1. 7 phosphoproteins including DEAD-box ATP-dependent RNA helicase, heat shock protein 90, Nuclear transcription factor Y subunit B, Eukaryotic translation initiation factor 4G, DnaJ homolog subfamily C member, CTR1, Putative LRR receptor-like serine/threonine-protein kinase were detected in both of studies.<br>2. DEAD-box ATP-dependent RNA helicase, heat shock protein 90 and CTR1 were up-regulated.<br>3. DnaJ homolog subfamily C member was down-regulated.	1. Cellular protein modif i cation, carbohydrate metabolism, and the nucleobase-containing compound process accounted for about 50% of all phosphoproteins in this reference.<br>2. Nuclear transcription factor Y subunit B-4 did not change significantly in this reference.<br>3. Eukaryotic translation initiation factor isoform 4G, Putative LRR receptor-like serine/threonine-protein kinase were up-regulated in this reference.	Comparative phosphoproteome analysis of the developing grains in bread wheat (*Triticum aestivum* L.) under well-watered and water deficit conditions
