# Supplementary figures and images for: Nitric Oxide Enhancing Resistance to PEG-Induced Water Deficiency is Associated with the Primary Photosynthesis Reaction in Triticum aestivum L
Source: Int J Mol Sci. 2018 Sep 18;19(9):2819. doi: 10.3390/ijms19092819 (PMC6164216; doi:10.3390/ijms19092819)

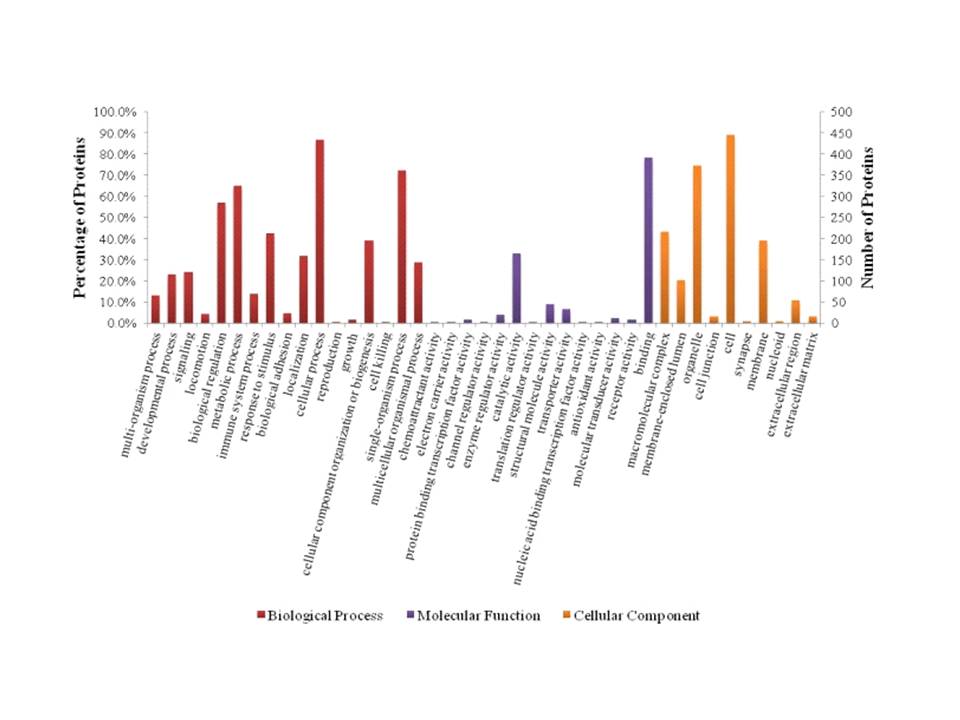

Supplement: Supplementary file 1 [file ijms-19-02819-s001.zip › Supplementary materials/Fig. S2.jpg]

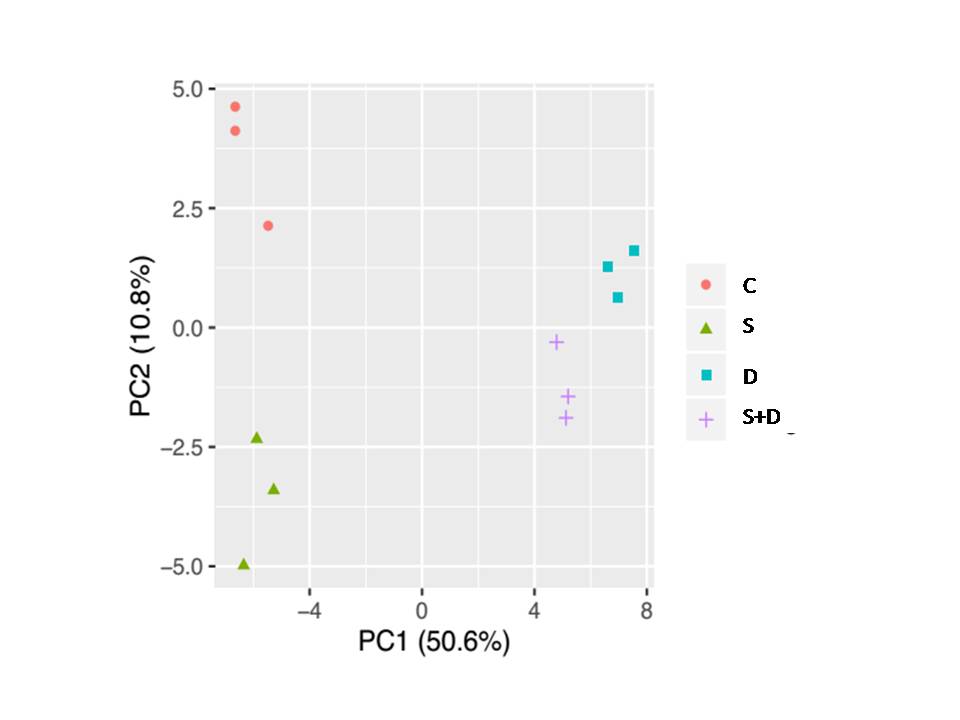

Supplement: Supplementary file 1 [file ijms-19-02819-s001.zip › Supplementary materials/Fig. S4.jpg]

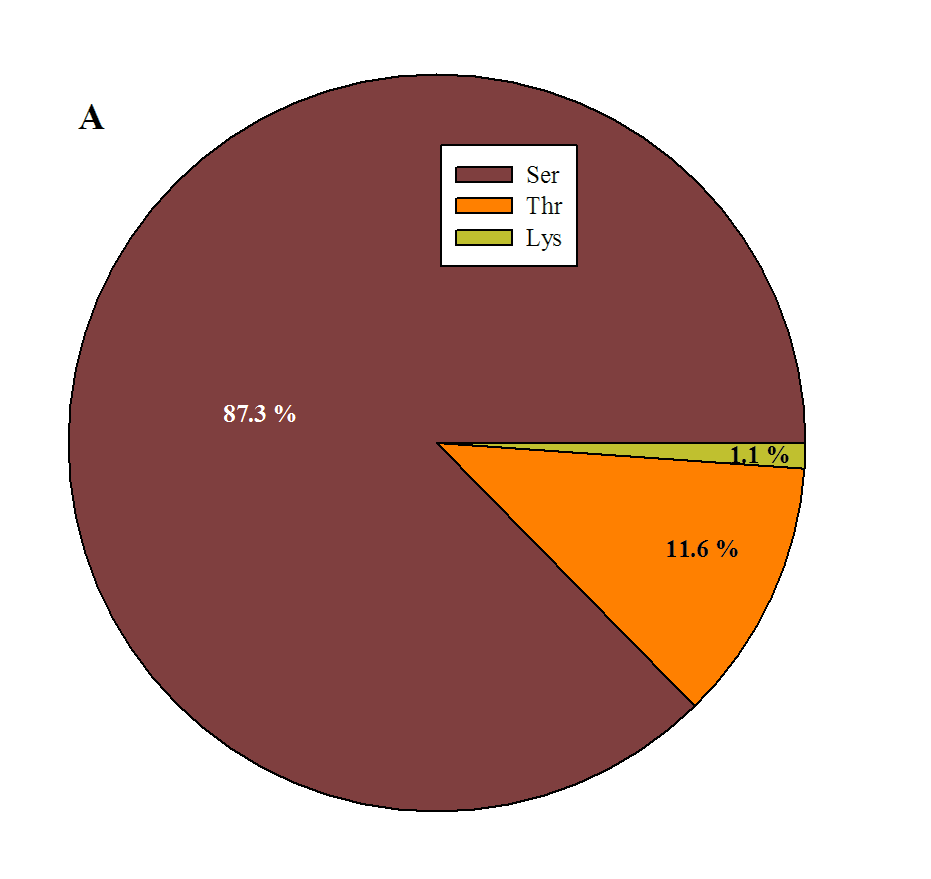

Supplement: Supplementary file 1 [file ijms-19-02819-s001.zip › Supplementary materials/Fig.S1A.TIF]

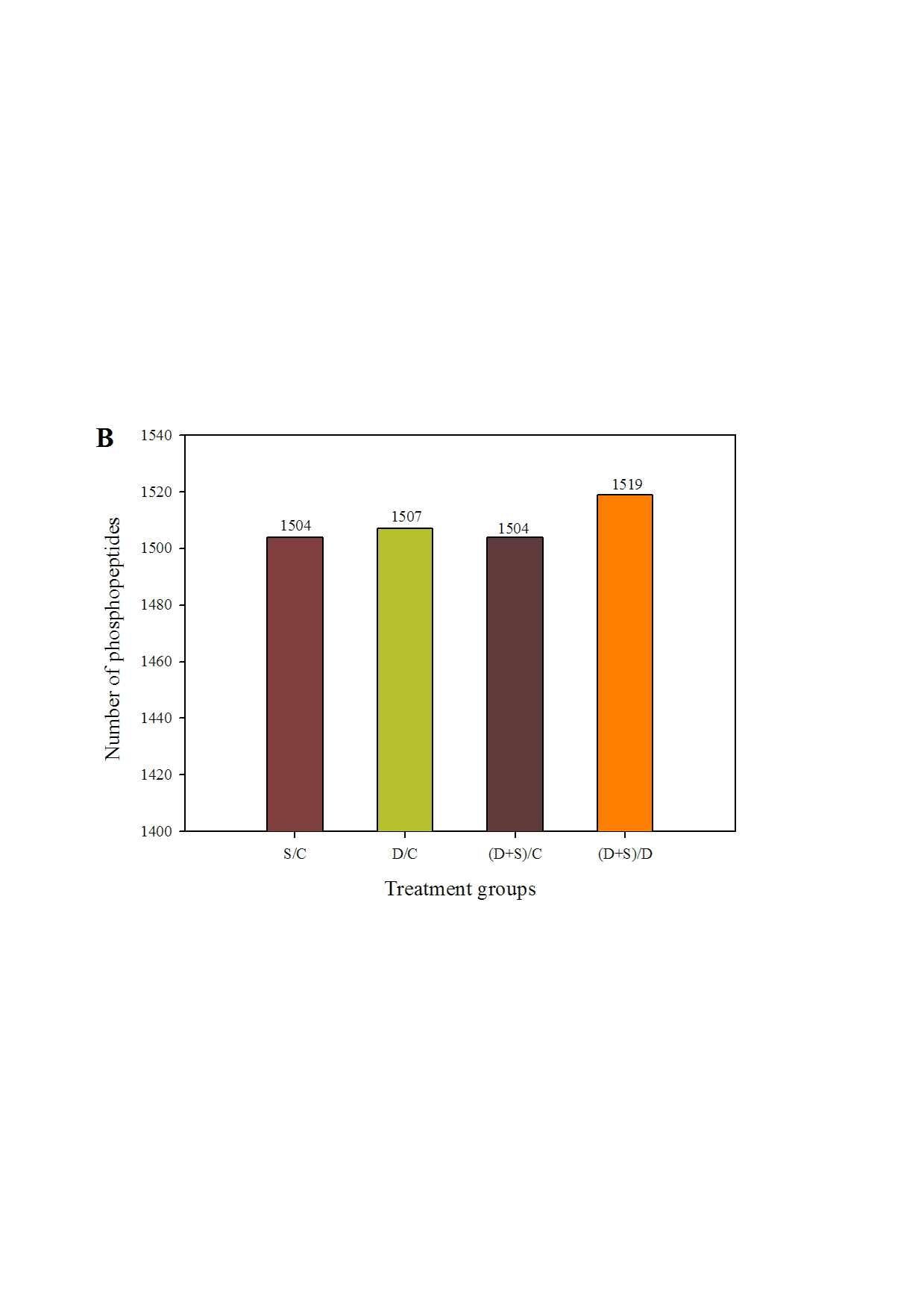

Supplement: Supplementary file 1 [file ijms-19-02819-s001.zip › Supplementary materials/Fig.S1B.TIF]

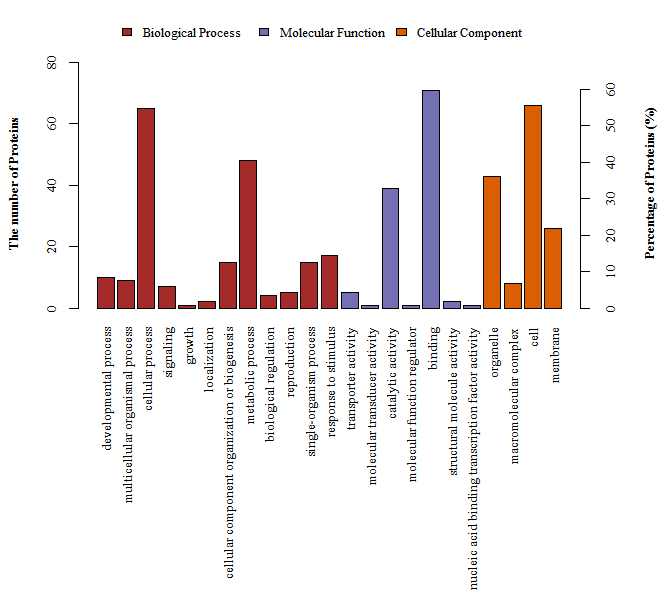

Supplement: Supplementary file 1 [file ijms-19-02819-s001.zip › Supplementary materials/Fig.S3A.tiff]

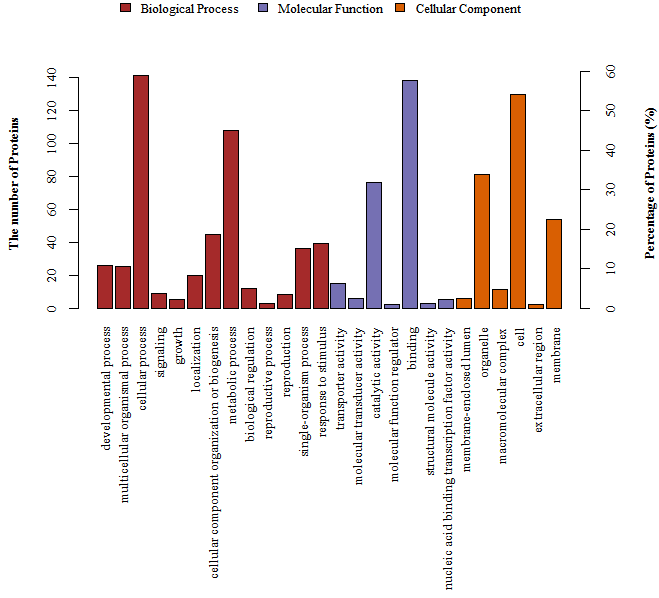

Supplement: Supplementary file 1 [file ijms-19-02819-s001.zip › Supplementary materials/Fig.S3B.tiff]

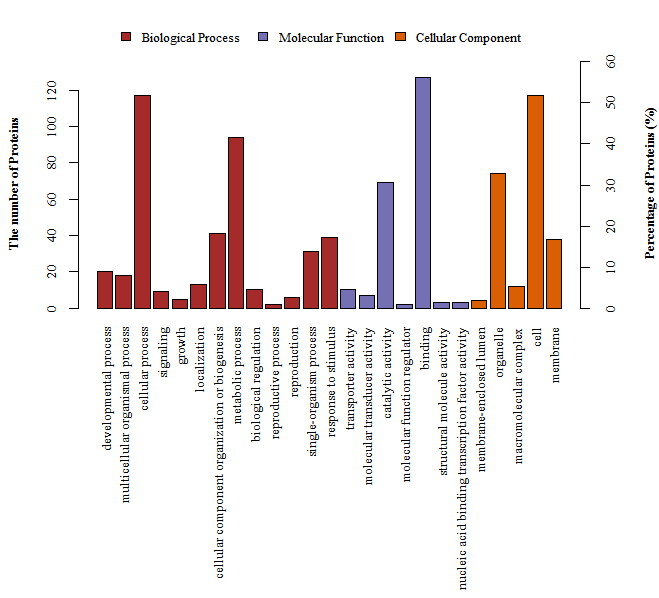

Supplement: Supplementary file 1 [file ijms-19-02819-s001.zip › Supplementary materials/Fig.S3C.tiff]

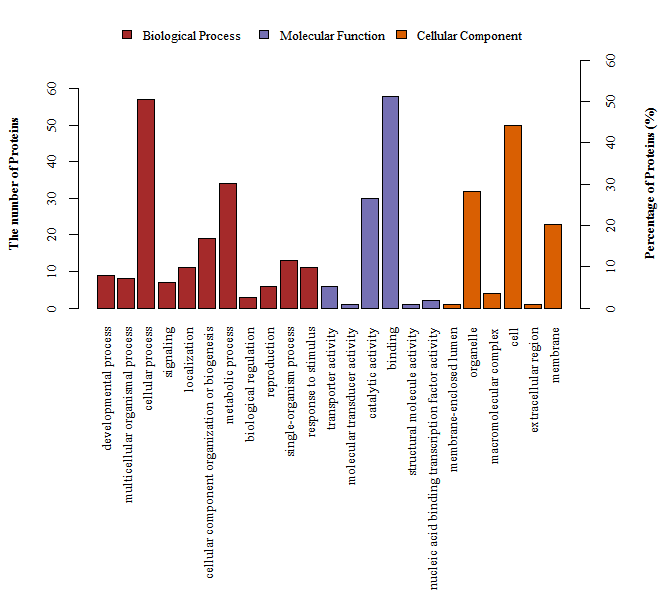

Supplement: Supplementary file 1 [file ijms-19-02819-s001.zip › Supplementary materials/Fig.S3D.tiff]
